# Artificial-Intelligence-Enhanced Analysis of In Vivo Confocal Microscopy in Corneal Diseases: A Review

**DOI:** 10.3390/diagnostics14070694

**Published:** 2024-03-26

**Authors:** Katarzyna Kryszan, Adam Wylęgała, Magdalena Kijonka, Patrycja Potrawa, Mateusz Walasz, Edward Wylęgała, Bogusława Orzechowska-Wylęgała

**Affiliations:** 1 Chair and Clinical Department of Ophthalmology, School of Medicine in Zabrze, Medical University of Silesia in Katowice, District Railway Hospital, 40-760 Katowice, Poland; adam.wylegala@gmail.com (A.W.); kijek303@gmail.com (M.K.); ewylegala@sum.edu.pl (E.W.); 2 Department of Ophthalmology, District Railway Hospital in Katowice, 40-760 Katowice, Poland; patrycja.potrawa@gmail.com (P.P.); m.walasz@rwdata.pl (M.W.); 3 Health Promotion and Obesity Management, Pathophysiology Department, Medical University of Silesia in Katowice, 40-752 Katowice, Poland; 4 Department of Pediatric Otolaryngology, Head and Neck Surgery, Chair of Pediatric Surgery, Medical University of Silesia, 40-760 Katowice, Poland; borzechowska-wylegala@sum.edu.pl

**Keywords:** artificial intelligence, deep learning, machine learning, in vivo confocal microscopy

## Abstract

Artificial intelligence (AI) has seen significant progress in medical diagnostics, particularly in image and video analysis. This review focuses on the application of AI in analyzing in vivo confocal microscopy (IVCM) images for corneal diseases. The cornea, as an exposed and delicate part of the body, necessitates the precise diagnoses of various conditions. Convolutional neural networks (CNNs), a key component of deep learning, are a powerful tool for image data analysis. This review highlights AI applications in diagnosing keratitis, dry eye disease, and diabetic corneal neuropathy. It discusses the potential of AI in detecting infectious agents, analyzing corneal nerve morphology, and identifying the subtle changes in nerve fiber characteristics in diabetic corneal neuropathy. However, challenges still remain, including limited datasets, overfitting, low-quality images, and unrepresentative training datasets. This review explores augmentation techniques and the importance of feature engineering to address these challenges. Despite the progress made, challenges are still present, such as the “black-box” nature of AI models and the need for explainable AI (XAI). Expanding datasets, fostering collaborative efforts, and developing user-friendly AI tools are crucial for enhancing the acceptance and integration of AI into clinical practice.

## 1. Introduction

Artificial intelligence (AI) is increasingly entering medicine all over the world. The approval of AI algorithms for the first time in healthcare use was in 1995, when Neuromedical Systems, Inc. (NSI) (Suffern, New York, NY, USA), developed the PAPNET^®^ Testing System to rescreen cervical smears. According to the data from 19 October 2023, the Food and Drug Administration (FDA) has approved approximately 700 AI algorithms for medical purposes since [1]. These mechanisms are mostly related to image and video analyses, and there is a great deal of information that can be extracted from an image using computer vision methods that the human eye may miss.

The cornea is a transparent part of the anterior segment of the eye that provides two-thirds of the eye’s focusing power and enables clear vision. However, as the first barrier against harmful environmental factors, it can be exposed to physical, chemical, and biological damage.

In vivo confocal microscopy (IVCM) captures cross-sectional images of the cornea with a thickness of several micrometers, which facilitates a noninvasive examination of every layer. Although artificial intelligence (AI) models have proven beneficial in various ophthalmological applications [2,3], the development of deep-learning-based systems in anterior eye segment diagnostics, especially when using IVCM images, still faces many challenges. A regular analysis of scientific reports and studies is crucial to enhance the awareness and understanding of AI as it enables subsequent investigators to achieve improved results in performance parameters, explainability, repeatability, and safety in their studies.

The aim of this review is to summarize and present the AI-assisted IVCM devices that have been developed over the last few years for keratitis, dry eye disease, and diabetic corneal neuropathy diagnostics.

## 2. Convolutional Neural Network Architecture

As is well known, convolutional neural networks (CNNs) are the most useful deep learning networks for image data analysis. To visualize what part of an image is important for classification, the algorithm learns by itself by relying on a large database of examples without human indication [4]. One of the goals of artificial intelligence is to enable machines to observe the world in a way that is similar to humans. This is possible through the use of neural networks. Neural networks are mathematical structures that are inspired by human neurons that are found in the brain. Their most common application is for image processing [5]. First, CNN models take an input image as an array of pixels, process it, and then finally classify it as a certain category [6].

A general model of CNNs consists of four components: the convolutional layer, pooling layer, activation function layer, and fully connected layer [7]. In the convolutional layer, the main mathematical task performed is called convolution. Convolution can be defined as a mathematical transformation of two functions that produces a third one that expresses how the shape of the first function is modified by the second [8]. An activation function is then applied (e.g., rectified linear unit activation function—ReLU or sigmoid function) after each convolution operation. This step enables the network to find nonlinear relationships between the features in the image [9]. Moving to the pooling layer, its goal is to reduce the dimensions of the feature arrays, which is what speeds up the computation process [10,11]. The fully connected layer represents the global information of the input object, and it also ultimately identifies to what class the image belongs [12]. At this stage, the activation function, when it is applied to the last fully connected layer, is used for a multiclass classification task. The most common one is the SoftMax function, which normalizes the output vector from the last fully connected layer to the probabilities of the target class (i.e., where each value ranges from 0 to 1 [13]).

## 3. Artificial Intelligence Issues

Due to the fact that there are incredibly few medical centers that collect data provided by confocal microscopy, there are still no publicly available materials that can be used to train a CNN. Deep learning methods have high accuracy when the amount of data is large for the purposes of training [14,15,16]. Although certain augmentation methods can improve model performance [17,18], models still benefit from having as big a dataset as possible.

### 3.1. Low Quality of Images

A classification process can be distorted by low-quality images [19,20,21]. Usually, bad images are excluded from a dataset such that they do not influence the final performance. Qu et al. [22] proposed a deep learning model to count and analyze the morphology of abnormal corneal endothelial cells at high noise levels and in poor-quality IVCM images. In reference to the first problem, which refers to the limited size of a dataset, it is important to recover as much data as possible from low-quality images.

### 3.2. Overfitting

The next common issue is overfitting. This problem means that the model only works well on the training data, but the generalization effect in testing is inaccurate [23,24]. It may be an effect of too many parameters being taken into account by the model in relation to the size of the training. Hence, it is better for the model to be less complex. Although it will achieve worse results on the training set, it will generalize the problem better and will classify new data more correctly. Another strategy to address overfitting is using a hold out or a cross-validation dataset [25,26]. Hold out is when one splits up a dataset into a “train” and “test” set [27]. A common split is using 80% of the data for training and 20% for testing; however, with respect to the articles analyzed in this review, 3:1 [28] and 10:1 [29] ratios have also been proposed. Cross-validation, on the other hand, divides the dataset randomly into “k” groups. One of the groups is used as the test set and the rest are used as the training set [30]. These are then partitioned multiple times until each group has been used as the test set [31].

If we cannot collect more diverse data, we can sometimes generate them ourselves. Although it sounds quite risky and dishonest, it is a common method in AI practice called augmentation. Data augmentation prevents the overfitting problem that was described above [32]. There is a great deal of room for improvement, especially in the area of image processing. We can slightly rotate the image, move it, change its colors, or make other more or less subtle changes that will give the model a significant amount of new data [33,34,35].

### 3.3. Unrepresentative Training Set

An unrepresentative training dataset is an issue that is similar to the overfitting problem. It generally means that the training data do not have enough diversity required, in one class, to properly train the model (e.g., the different phenotypes of corneal bacterial infection in IVCM images). It is, thus, recommended to collect variant features that are the least represented in the training data [36,37]. This is where the process called feature engineering plays a significant role. It consists of selecting the most useful features (among the available features) and feature discovery (combining the existing features to obtain more useful features) [38,39].

### 3.4. Limited Dataset Size

N-shot learning (NSL) proves advantageous in scenarios involving challenging images, especially when dealing with limited training data. In this context, a “shot” denotes a single example that is available for training, and “N” represents the number of these examples. NSL is broadly defined and has the following subfields: zero-shot learning, one-shot learning, and few-shot learning. The basic idea of zero-shot learning is to use the model’s existing knowledge (which is usually based on a set of provided examples, e.g., appearance, proportions, or functionality) to classify new data that have not been encountered before [40]. One-shot learning allows a model to learn from a single data example. Few-shot learning is similar to one-shot learning except that it has more than one training example to learn from. According to the few-shot learning approach, which usually means N-way-K-shot classification (where N stands for the number of classes and K for the number of examples from each class), the main task is to classify the “Q”-query images among the N classes.

### 3.5. “Black-Box” Problem

One of the significant obstacles faced by scientists implementing program solutions based on artificial intelligence is the lack of social acceptance and trust. The term “black-box” in the context of AI means that artificial intelligence does not contain information about how it achieved its results [41]. Due to the problems of the “black-box”, the explainable AI (XAI) approach was established. XAI can be understood as methods that will enable humans to understand the output produced by machine learning algorithms. The use of saliency maps indicates the areas of the image that have the greatest impact on prediction, i.e., what increases usefulness and the understanding of users. A saliency map is an image segmentation method that analyzes every pixel and gives it a validity label in the output classification [42,43].

## 4. Methods and Materials

The articles were collected through PubMed, and the appropriate publications were analyzed in review. All of the available studies that focused on artificial intelligence in confocal microscopy were included. The main purpose of this review was to present the usefulness of clinical applications analyzing IVCM images in ocular surface disorders such as keratitis, dry eye disease, and diabetic corneal neuropathy. Criteria and a search strategy were established. All articles were found in the PubMed database. The search keywords included “artificial intelligence/AI”, “confocal microscopy/IVCM”, “deep learning/DL”, and “machine learning/ML”. When we take a look into the past, we can note that artificial intelligence has developed significantly over the last 5 years. Furthermore, in the field of ophthalmology, 2020 was a real breakthrough year in terms of the amount of research publications related to machine learning. The main timeline was set from 2018 to 2023, but we also strived to use the most recent papers. The reason for this was the desire to share the latest knowledge and scientific reports, which—while these studies often use mechanisms discovered by their predecessors—are now being upgraded with new methods that improve their effectiveness and efficiency. Only original research articles written in English were included, i.e., reviews, editorials, opinions, single case reports, and ex vivo studies were excluded. The reference lists of the remaining studies were also checked, and they served as supplementary literature in the review. Publishers were scrutinized and the preference was given to peer-reviewed, academic journals and reputable websites. Our primary objective was to mitigate potential biases and to specifically address the risk of content omissions and unnecessary overlapping. Each article underwent individual assessments for coherence, completeness, and scope. This was achieved through a rigorous analysis of results, including performance outcomes, limitations, and future solutions. Constructive feedback from all authors was conducted.

We excluded articles where no artificial intelligence networks were mentioned. Studies where recovery of the full text was not possible, even after searching the available medical databases, were also excluded. A total of 34 articles were included in the final manuscript. Following this initial phase, the manuscript underwent iterative refinement. The revised versions were once again subjected to a thorough review by all authors, with subsequent amendments overseen by the first author. The final iterations were then circulated to the senior author for their meticulous evaluation and ultimate approval.

**Figure 1:**
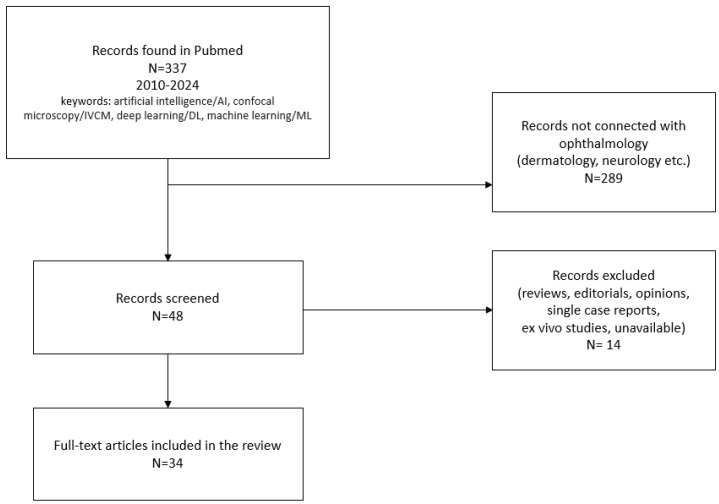
Flowchart of the literature search history.

A PRISMA flow diagram to visually depict the systematic inclusion of articles was incorporated (see Figure 1 for the article selection process in detail).

## 5. Evaluation of Individual Disease Articles

This section is divided into three separate parts, each of which involves the review of articles by analyzing different diseases—keratitis, dry eye disease, and diabetic corneal neuropathy.

### 5.1. Keratitis

Infectious keratitis is caused by microorganisms such as bacteria, fungi, protozoa, and viruses [44]. The most common cause of keratitis is a disruption of the corneal epithelium, which serves as an excellent passage for microorganisms. After cornea penetration, the anterior chamber inflammation starts with acute and severe pain. Without early and proper treatment, it may lead to subsequent vision loss, infection of the posterior segment of the eye, and need for surgery [45,46]. The gold standard in the diagnosis of keratitis still remains microbial culture [47]. However, IVCM might serve as an additional useful diagnostic tool, and it may also help in implementing empirical treatment as soon as possible.

#### 5.1.1. Fungal Keratitis

Mycotic keratitis is one of the most severe inflammations of the cornea. It can be observed worldwide, with increased frequency in tropical and subtropical areas [48]. Certain risk factors can be defined, such as contact with agriculture, the use of corticosteroids, and the use of contact lenses, as well as systemic diseases like diabetes mellitus or immunosuppression. In IVCM images, fungi usually show up as linear, branching, and hyperreflective filaments. Their filament diameters vary between 1.5–7.8 μm, and the total length can reach up to 400 μm [49].

**Figure 2:**
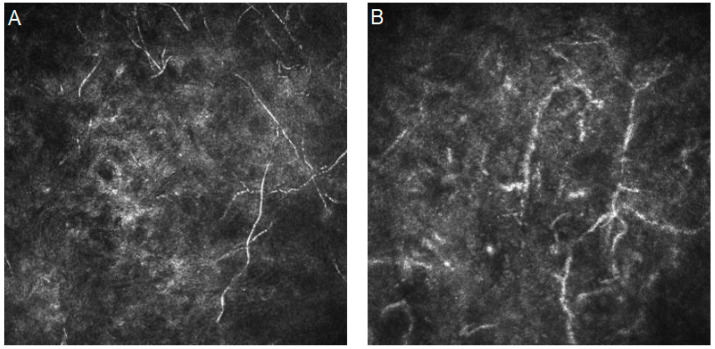
In vivo confocal microscopy images of fungal keratitis. (**A**,**B**) Bunches of hyper-reflective, linear structures with acute angle branching. Resolution 400 × 400 µm.

Figure 2 presents confocal images with mycotic keratitis characteristics.

#### 5.1.2. Bacterial Keratitis

The severity of bacterial keratitis varies depending on geography, climate, national development, and access to medical care. The main risk factors include ocular surface diseases, contact lens wear, systemic immunosuppression, prior corneal surgery, use of topical steroids, and trauma [50]. Although bacteria, except for *Nocardia* spp., are often too small to be detected as visible structures by confocal microscopy [51], there are certain premises that may indicate bacterial infection, such as an abundance of polymorphic neutrophils or a lack of atypical elements [52].

**Figure 3:**
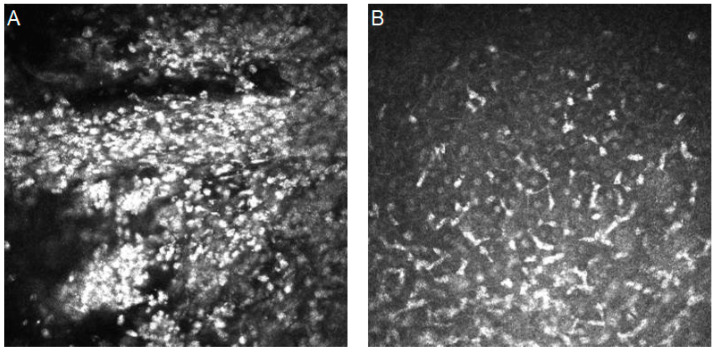
In vivo confocal microscopy images of bacterial keratitis. (**A**,**B**) No atypical organisms such as Acanthamoeba, fungal filaments, or yeasts. The presence of A means a significant influx of leukocytes, and the presence of B means “dendritiform” cells and keratocyte activation. Resolution 400 × 400 µm.

Figure 3 shows confocal images with bacterial keratitis characteristics.

#### 5.1.3. Acanthamoeba Keratitis

Acanthamoeba is a group of protozoa that live in the form of cysts and trophozoites. They can be found in many water sources, both potable and nonpotable. The majority of cases are connected with the use of contact lenses [53,54]. In IVCM images, they are usually found as highly reflective oval cysts surrounded by a low-refractile wall that has a clear boundary and a dark ring outside [55].

**Figure 4:**
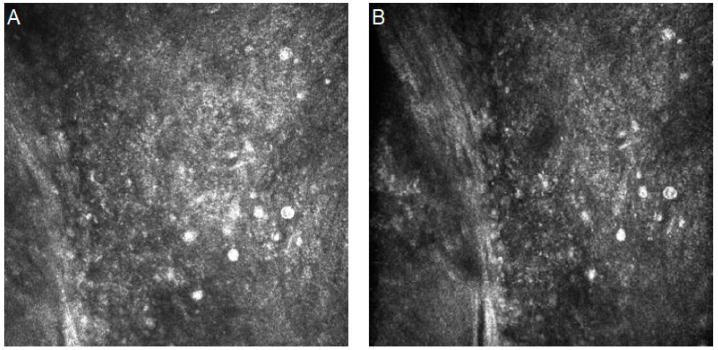
In vivo confocal microscopy images of Acanthamoeba keratitis. (**A**,**B**) Highly reflective oval cysts with a low-refractile wall that has a clear boundary and a dark ring outside. Resolution 400 × 400 µm.

Figure 4 shows confocal images with Acanthamoeba keratitis characteristics.

#### 5.1.4. Viral Keratitis

Although many viruses have been shown to cause keratitis, the herpes viruses are the prevailing etiological cause of viral keratitis [56]. The herpes simplex virus can affect all layers of the cornea [57]. IVCM findings regarding this include the following: the presence of hyperreflective and irregular epithelial cells; Langerhans cells within the basal epithelium layer [58]; and a decreased number of sub-basal nerves and increased tortuosity. Changes in the nerve characteristics have also been observed in the herpes zoster virus [59]. Mangan et al. [60] presented a patient with corneal irregular epithelial cells, scattered inflammatory cells and cell debris, and activated dendritic cells in the sub-basal epithelial area with a marked decrease in the sub-basal corneal nerve plex during COVID-19 infection. Adenoviruses can occur as cell clusters in the basal epithelial layer with increased Langerhans cell presence and hyperreflectivity areas in the anterior stroma [61].

**Figure 5:**
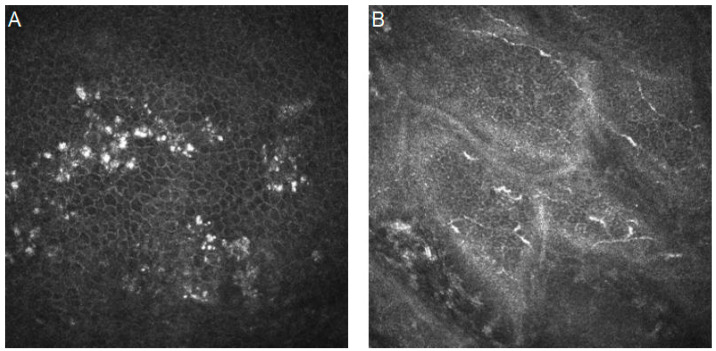
In vivo confocal microscopy images of herpes zoster keratitis. (**A**) Highly reflective keratic precipitates. (**B**) A decrease in nerve length and the presence of dendritic inflammatory cells. Resolution 400 × 400 µm.

Figure 5 shows confocal images with viral keratitis characteristics.

A manual analysis of IVCM keratitis images requires specialized staff, as well as a significant amount of time to properly examine each case. AI is increasingly contributing to speeding up and improving the overall accuracy of diagnostics. Below, the selected articles will be discussed.

Essalat et al. [28] tested eight deep learning models based on convolutional neural networks (CNNs) to create automated support in the diagnostic accuracy of confocal microscopy of infectious keratitis. The dataset was divided into four groups: Acanthamoeba keratitis, fungal keratitis, nonspecific keratitis, and healthy eyes. The best model (Densenet161) achieved an accuracy, precision, recall, and F1 score of 93.55%, 92.52%, 94.77%, and 96.93%, respectively. These performance metrics are usually used to describe the performance of medical devices [62]. The authors emphasized that their proposed algorithm can help ophthalmologists provide faster and more reliable diagnoses by implementing a saliency map that highlights infectious areas in the IVCM images.

Alisa Lincke et al. [63] proposed an AI-based decision support system for the automated diagnosis of Acanthamoeba keratitis (AK). They used ResNet101V2 with transfer learning implementation. Despite the low sensitivity of the AK diagnosis (16.6% of correct model’s predictions), their proposed system reduced the time needed to sort and analyze IVCM images, which was achieved by dividing them into healthy and unhealthy ones.

Xuelian Wu et al. [64] compared the automatic hyphae detection and quantitative evaluation of confocal images with corneal smear results. The accuracy of their proposed technology was better than current corneal smear examinations (*p*  <  0.05).

Shanshan Liang et al. [65] used a two-stream convolutional network—GoogLeNet and VGGNet—to diagnose fungal keratitis. The main stream is used for extracting the important parts (i.e., groups of pixels) of the input image. The second stream is used for discriminating between the background and the intensified pixels that create the hyphae. The dual-stream structure allows users to have more influence over the segmentation results. Moreover, it provides a better performance compared to the single-stream networks. The features extracted by every stream were integrated to perform the final prediction. The proposed model resulted in an accuracy, precision, sensitivity, specificity, and F1 score of 97.73%, 98.68%, 97.02%, 98.54%, and 97.84%, respectively.

Jian Lv et al. [66] also based their approach on images of patients with confirmed fungal infection via fungal culture. In the testing dataset, their ResNet101 CNN model showed an accuracy of 96.26%, a specificity of 98.34%, and a sensitivity of 91.86%. What was interesting, however, was that, after adding diabetic patients into the training set, the accuracy decreased to 93.64%, the specificity increased to 98.89%, and the sensitivity decreased to 82.56%. The reason for this may lie in the reduction in nerve fibers in the corneal tissue of diabetic patients [67]. Apart from the worse results, it was found to be more realistic because diabetic patients are common patients in the clinic. In their next work [68], they paid attention to comparing the performance of ophthalmologists who were assisted by the black-box AI model and the explainable AI model (XAI) in terms of diagnosing fungal keratitis. The explainable model consisted of histograms showing the model prediction probabilities of positive and negative fungal keratitis presence. Overall, the performance in the XAI-assisted diagnostics was better than the AI-assisted diagnostics, and both tools produced better performances than the work that was conducted without their assistance. This effect was more evident for inexperienced doctors compared to experienced doctors. Another interesting observation was made—although the time with the XAI-assisted device was higher than that without assistance, the difference was not statistically significant (*p* = 0.092). This situation shows that people involved in science and medicine not only want to make their work easier and more efficient with new tools, but they also wish to understand how these tools work.

Certain attempts were made not only to detect fungal hyphae but also to recognize their species. Ningning Tang et al. [69] designed an automated method to distinguish Fusarium and Aspergillus genres. To cope with the overfitting phenomenon, they used transfer learning to improve their model’s generalization ability. Transfer learning is an approach to machine learning that involves using the knowledge acquired while solving one task and then applying it to perform another (which is relatively similar in the field) [70]. In this study, the datasets were determined according to the microbiological culture results, which are, in actuality, not possible for humans to recognize. The models were valid in their judgments with an area under the curve (AUC) of 88.7% for Fusarium and an AUC of 82.7% for Aspergillus.

Ningning Tang et al.’s [71] project was based on dual hybrid systems that were aimed at the automated identification of corneal layers from IVCM images. They developed two classifiers based on CNNs and KNNs (K-neighbor networks). The first one was used to analyze the pixel information, and the other one was used to analyze the scanning depth information. Then, two hybrid strategies (a weighted voting method and the LightGBM algorithm) collected the outputs of the two base classifiers. A weighted voting method gained the best classification result. Both hybrid approaches achieved better performances when compared with the CNN or the KNN alone.

Zhi Liu et al. [29] trained a novel CNN that uses data augmentation and image fusion to detect fungal keratitis. In this work, normal images were augmented by image turnovers. That increased the number of corneal images from 219 to 876. The novel SCS method, which is based on CS (contrast stretching), was also used to preprocess the original image to highlight important features without an information loss. Then, the fusion was conducted. They improved the basic method called MF (mean fusion), which is an approach that relies on matching the images of the same size and taking the average [72]. HMF (histogram mean fusion) is used to create an image histogram that represents the grayscale of the image. Accordingly, the grayscale of the preprocessed SCS-based image matches the original image. In this way, the merged image has the same gray level as the original image, while the distinction of key structures in the SCS-based preprocessed image is preserved. This experiment of combined CNNs—AlexNet and VGGNet—using histogram matching fusion (HMF) achieved an accuracy of 99.95% and 99.89%, respectively. Moreover, compared to the traditional AlexNet and VGGNet, it was 99.35% and 99.14%, respectively.

Fan Xu et al. [73] proposed a deep transfer learning model, Inception-ResNet, for the detection of activated dendritic cells and inflammatory cells with high accuracy. The dataset included patients with keratitis, dry eyes disease, and pterygium. The accuracy of the model was similar to an experienced ophthalmologist and better than a beginner ophthalmologist.

Yulin Yan et al. [74] worked on an automatic mechanism for the fast recognition of the layers of corneal images using in vivo confocal microscopy. Additionally, they differentiated them as normal and abnormal. The abnormal images included cases of the edema of epithelial cells, enlarged interstitial spaces, inflammatory cells, nerve fiber tortuosity or thinning, Langerhans cell amounts, stromal swelling, scarring, pathogen infiltrations (Acathamoeba, fungi), neovascularization, and endothelial cell swelling or deposits. A comparison between humans and machines showed that the model was as accurate as an experienced ophthalmologist and about 237 times faster than a human. At the same time, the accuracy of inexperienced doctors in IVCM image recognition using the model could be significantly improved and may even approach that of specialists. The author paid attention to dataset volume and encouraged readers to develop interhospital cooperation to expand the databases for more detailed analyses.

Table 1 summarizes the articles described above.

### 5.2. Dry Eye Disease

Dry eye disease (DED) is a consequence of insufficient ocular surface moisture via the tear film. It may be caused by the composition of incorrect layers or excessive evaporation. It is often associated with autoimmune diseases (e.g., rheumatoid arthritis, Sjögren’s syndrome [75,76,77], Graves–Basedow disease [78], graft versus host disease [79]), as well as dermatological (e.g., pemphigoid [80] and rosacea [81]) and neurological diseases (e.g., Parkinson’s disease [82] and Bell’s palsy [83]). It may occur postoperatively, e.g., after laser refractive procedures [84], after cataract surgery [85], after the use of drugs (e.g., antihistamines, antidepressants, and contraceptives), and in cigarette smokers [86].

In dry eye syndrome, the following IVCM image features have been reported [87]: a decreased density in the corneal superficial epithelial cell, increased density in the corneal anterior keratocyte, increased density in the inflammatory dendritic cell, a decreased number of sub-basal nerves, and increased nerve tortuosity.

**Figure 6:**
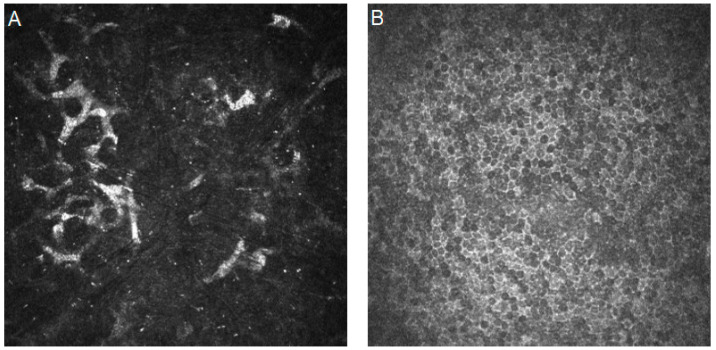
In vivo confocal microscopy images of dry eye disease. (**A**) An increased density of highly reflective keratocytes. (**B**) A decreased density of corneal epithelial cells. Resolution 400 × 400 µm.

Figure 6 shows confocal images of dry eye disease characteristics.

Shanshan Wei et al. [88] designed a deep learning model called CNS-Net, which was designed to analyze sub-basal nerve morphologies. It allowed for the possibility of obtaining the average density and the maximum length of the nerve fiber with a high accuracy that produced an AUC of 96%. The model can also analyze 32 images per second, a feat that is practically impossible for a human, and it was able to ensure that nerve fibers were not missed when compared to an ophthalmologist.

Dalan Jing et al. [89] used the previously mentioned CNS-Net model to study the relationships between corneal sub-basal nerve parameters and corneal aberrations in dry eye disease. The ocular surface irritation pain was found to be positively interrelated with anterior corneal aberration. In their next study [90], the same algorithm measured sub-basal nerve parameters to investigate the association between oval cells, Langerhans cells (LCs), and dry eye disease. In opposition to the studies [91,92] showing a decrease in the nerve length, it was observed that—with the presence of the LCs and bright, oval cells—there was a greater corneal peripheral nerve maximum length and average density. This suggests that the changes in corneal nerve density and nerve number are related to the level of advancement of the dry eye disease. It can, therefore, be concluded that an increased length and number of nerves occur in mild and intermediate dry eye conditions.

Gairik Kundu et al. [93] investigated corneal nerve characteristics using confocal microscopy in patients presenting ocular surface pain. Orthoptic-related issues and systemic diseases were also included in the artificial intelligence algorithm, which operated on a random forest (RF) classifier. One of the key advantages of random forest is its ability to handle large and complex datasets. Microneuromas were defined as the parameter with highest importance by the RF model, and they could also be the possible reason for the pain.

An objective tool for sub-basal plexus nerve tortuosity level determination was designed. Tortuosity grading delivers information about corneal nerve reconstruction—a vicious circle of degeneration and regeneration processes. Yitian Zhao et al.’s [94] 2020 device CS-NET, which is based on the Retinex model [95], was used to enable image quality enhancement. The grading of the tortuosity level was achieved with the linear support vector machine. With their AI model, Baikai Ma et al. [96] suggested that cornea nerve tortuosity is a potential biomarker for corneal neurobiology in dry eye disease. Fernández, I. et al. [97] investigated post-LASIK dry eye syndrome and noted an increased nerve tortuosity compared with the control group. 

Ye-Ye Zhang et al. [98] and Sachiko Maruoka et al. [99] investigated meibomian glands. Meibomian gland dysfunction (MGD) can lead to a decreased or decomposed tear film lipid layer and inflammation, which causes dry eye disease [100]. Zhang et al. [98] trained three types of convolutional neural networks to differentiate the meibomian gland appearances. Among them, the DenseNet169 network showed the highest accuracy of 97.3% in obstructive MGD (OMGD), 98.6% in atrophic MGD (AMGD), and 98% in the healthy controls. It was better than an ophthalmologist’s accuracy of 91%. Maruoka et al.’s [99]. DenseNet-201-based model achieved an area under the curve, sensitivity, and specificity for diagnosing obstructive MGD at 0.966%, 94.2%, and 82.1%, respectively. In addition, for the ensemble various DL model, it achieved values of 0.981%, 92.1%, and 98.8%, respectively.

Harry Levine et al. [101] presented an automated algorithm for the detection of dendritic cells in the IVCM images of central corneas. Despite obtaining slightly worse algorithm results compared to the manual counts, the authors suggested that the further development of this algorithm can improve generalizability and performance.

Md Asif Khan Setu et al. [102] analyzed both dendritic cells and corneal nerve fibers using U-Net CNN and Mask R-CNN architectures. The proposed model was able to segmentate nerve fibers, define nerve tortuosity, count total nerve density, and punctate branch points, all in combination with dendritic cell detection (an objective tool that was created to differentiate the severity of ocular surface disorder).

Table 2 summarizes the articles described above.

### 5.3. Diabetic Corneal Neuropathy

Diabetic corneal neuropathy is one of the most common ocular complications in diabetes. The cause of diabetic neuropathy is high blood glucose levels, which results in the formation of the glycation end products that result in changes in the nerves [103]. IVCM images can show the curtailment of corneal nerve fiber lengths, as well as nerve fiber density reduction, nerve fiber branch density dilution, and increases in nerve fiber tortuosity [104].

**Figure 7:**
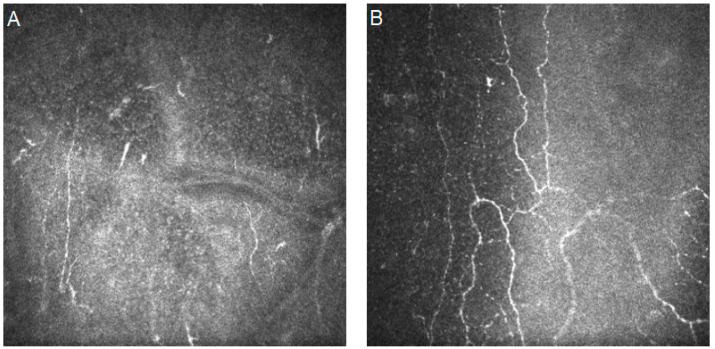
In vivo confocal microscopy images of diabetic corneal neuropathy (patient with diabetes type 1). (**A**) Decreased nerve fiber lengths and densities. (**B**) Increase in nerve fiber tortuosity. Resolution 400 × 400 µm.

Figure 7 shows confocal images with diabetic corneal neuropathy characteristics.

The first steps in automated nerve fiber detection were taken by Dabbah et al. in 2010 [105] and 2011 [106], as well as Petropoulos et al. (2014) [107], who relied on 2D Gabor filters and Gaussian envelopes. The Gabor filter is a linear filter used for texture analysis. It determines any particular and regular feature in the image in a localized area around the place of analysis in a specific line-based way. Then, these methods were improved by Xin Chen et. al. [108] in terms of sensitivity and of accuracy 0.917 and 0.913, respectively. A similar method [109], called the corneal nerve fiber fractal dimension, was then used for automated measurements of corneal nerve complexity.

Wei Tang et al. [110] proposed a multiscale feature guidance neural network (MLFGNet) for automatic corneal nerve fiber segmentations in IVCM images. In the literature, it was found that multiscale feature fusion can improve the detection accuracy of all kinds of objects, including objects with a relatively small scale [111] (in this case, e.g., thin nerve fibers). This novel deep learning instrument observes the information aggregation from high-level features to low-level features, and it also reduces the information gap between different levels. The model even captured the curvilinear structure of nerve fibers while the other methods did not.

Tooba Salahouddin et al. [112] proposed a model, based on the U-Net network and an adaptive neuro-fuzzy inference system, which differentiates diabetic peripheral neuropathy. It allows one to compare each class with one another. The classification of diabetic peripheral neuropathy presence in diabetic patients achieved a 92% score in sensitivity and 80% in specificity. Furthermore, the model detected corneal nerve damage in patients who were understood to have no diabetic peripheral neuropathy in terms of their Toronto Clinical Neuropathy Score. Thus, the model could be considered a more sensitive approach for early small nerve pathologies.

Yanda Meng et al. [113] modified their previous AI-based algorithm [114] to classify patients with prediabetes and diabetes into those with or without peripheral neuropathy. There was no need for expert annotation, and the algorithm had a sensitivity of 91% and a specificity of 93% in detecting nerve fiber disorders. This type of algorithm prototype could serve as a rapid, automated screening tool through which to provide neuropathy detection.

The algorithm prepared by Williams et al. [115] was trained on 1698 corneal confocal microscopy images. It was then tested on 2137 images, both containing healthy controls or certain nondiabetes conditions (e.g., keratoconus and pseudoexfoliation syndrome) and diabetic participants. The algorithm, which is based on the U-Net network, identified the total nerve fiber length, branch points, and tail points, as well as the number and length of the nerve segments. It was then compared with the widely used and validated automated image analysis software ACCMetrics (Version 2.0, Early Neuropathy Assessment [ENA] group, University of Manchester, Manchester, UK), and it was found to perform better with the analyzed parameters.

Erdost Yıldız et al. [116] confronted GAN-based algorithms with U-Net algorithms, which aim to automatically segment the corneal sub-basal nerves in IVCM images. It is interesting that the authors added noise to the images to simulate everyday challenges in ophthalmology clinics, as well as lowered image quality to verify whether the algorithms could still work properly. 

NerveStitcher, which was designed by Guangxu Li et al. [117], is a novel stitching framework that is based on a convolutional neural network and a graph convolutional neural network. It enables the merging of multiple images with overlapping fields of view to create a larger mosaic-like image with a wider field of view. This then provides an opportunity through which to retrace the nerve length and morphology in larger areas.

An interesting direction taken by Abdulhakim Elbita et al. [118] was the idea of creating 3D corneal layer models that could provide a better visualization of the anatomy as well as a more accurate identification of diseased locations. 

Table 3 summarizes the articles described above.

## 6. Conclusions

It is clear that artificial intelligence will become a permanent fixture in medicine, and existing algorithms will be continuously improved upon to meet the most pressing needs of doctors and patients. To ensure better clinical compliance, AI devices should be interpretable and understandable to clinicians, as they should support their diagnostic and therapeutic decisions.

A manual analysis of IVCM images is time-consuming, even if performed by an experienced ophthalmology specialist. The automation of this process is needed and is necessary to address shortages of qualified staff, speed up diagnostics, and reduce treatment costs. Moreover, it is worth observing the latest studies and solutions to explore the performance of combined deep learning methods. 

Comparing the results of analyses performed via a computer with the work of a human may be controversial, but we should look at it in a different way. AI is not supposed to replace humans; rather, its goal should be to help humans achieve better results at work and to be more effective with less energy wasted. DL models can quickly filter a large amount of data and exclude images of healthy cases: this allows them to provide ophthalmologists with the images that most likely represent infectious structures (along with the model’s diagnosis and confidence level). This process can significantly reduce the workload of ophthalmologists and IVCM technicians. It can also serve as a second independent opinion that supports less-experienced doctors and improves their self-assurance in diagnoses.

CNNs are the most useful and comprehensive deep learning networks for image data analysis, despite the many challenges and difficulties faced by artificial intelligence algorithms. Some of these aforementioned issues have already been resolved, but we should still strive for solutions that are explainable and clear to us in terms of algorithmic decision interpretation. Explainable systems could teach IVCM analysis skills in places where there is a shortage of well-qualified medical staff. Explanatory maps can quickly help indicate the most important features that can guide further diagnostics, thereby reducing test time and associated costs.

Combining IVCM with other examination methods, such as slit-lamp microscopy images or optical coherence tomography (OCT) scan results, could create a comprehensive diagnostic device for better disease management.

Despite the increasing amount of research on artificial intelligence in the diagnostics of the anterior segment of the eye, there is still a lack of research on bacterial keratitis. The cause for this may be found in its size being below the resolution that IVCM images can capture. In addition, it is not possible to discriminate bacterial species in IVCM images. There are also no studies that are directly related to analyzing viral diseases in IVCM images. This may be due to the fact that they are perceived as neurotrophic inflammation and are diagnosed on the basis of nerve parameter changes (neurotrophic keratopathy) instead. 

Mostly due to the secondary etiological character of dry eye disease, a multidisciplinary approach is required, and, consequently, a patient’s willingness to engage in the diagnostic and therapeutic process. This could be a problem for elderly, sick people, and we should meet this challenge by shortening the time needed for accurate diagnoses via the implementation of AI-powered devices in our work.

Recently, the Food and Drug Administration (FDA) approved the first autonomous AI-based DL algorithm to screen for diabetic retinopathy. Thanks to automated detection and the characteristics of corneal nerve fibers, there are opportunities on the horizon for screening devices that can detect early neuropathy. Such a device could lead to the effective prevention of advanced diabetic neuropathy complications via allowing patients to receive early professional treatment. It is worth mentioning that further exploration of cost-effective models needs to be executed to assess their influence on health economics. 

It is crucial to design high-quality enhancements of the captured images, i.e., contrast intensification, such that the algorithm does not overlook thin and faint nerve fibers (which are the fibers that are first affected in diabetic corneal neuropathy). Moreover, diabetic corneal neuropathy should be always considered with systemic complications, and glucose level blood tests should be used for better disease management.

## 7. Future Directions

It is crucial to constantly improve the performance of AI-supported devices to render their predictions even more accurate and to enable the possibility of working with various types of data. New and advanced algorithms should be utilized to protect data size, which would therefore lead to analyzing the unfocused and poor-quality images that are often captured by the IVCM method. AI devices can be applied to hospitals that have little clinical experience or have shortages of qualified staff. We encourage others to purchase IVCM devices, even without experienced IVCM interpreters, in order to explore their usefulness in automatic image analysis. 

For all the diseases presented, a limitation was found in that the studies did not include any measurements of parameter change but instead looked at a single time point. This may be a clue for future researchers in terms of analyzing the performance of model interpretations during disease progression. In the future, we would like artificial intelligence to be able to not only recognize the disease but also to determine its severity and treatment response.

It is crucial to share multicenter studies that encompass larger, different ethnic groups in order to establish a clinically reliable algorithm that could be used anywhere in the world.

With increasing model generalization, an intelligent screening of corneal diseases will be possible. Having said this, we should not only be concerned about detection pathologies but also their grading and treatment. It could be that, in the future, AI will give us an answer regarding the necessity of treatment, as well as its expected effectiveness and side effects.

## Figures and Tables

**Table 1:** Summary table for the different DL systems in the detection of keratitis when using IVCM. The performance parameter types depended on the authors’ choice and show the results of the best proposed approaches. Additional techniques and novelties are featured.

Authors	Year	Dataset	Artificial Intelligence Method	Results		Additional Techniques and Novelties
Essalat et al. [28]	2023	4001 images	CNN—Densenet161	Accuracy 93.55% Precision 92.52% Recall 94.77% F1 score 96.93%		Saliency maps.
Alisa Lincke et al. [63].	2023	68,970 images	CNN—ResNet101V2	Healthy/diseased—95% accuracy		Transfer learning.
Xuelian Wu et al. [64]	2017	82 patients	Adaptive robust binary pattern	The accuracy of the model was superior to the corneal smear examination (*p* < 0.05) approach.		Support vector machine.
				Sensitivity 89.29%		
				Specificity 95.65%		
				AUC 0.946		
Shanshan Liang et al. [55]	2023	7278 images	SACNN—GoogLeNet and VGGNet	Accuracy 97.73%		Two-stream convolutional network.
				Precision 98.68%		
				Sensitivity 97.02%		
				Specificity 98.54%,		
				F1 score 97.84%		
Jian Lv et al. [66]	2020	2088 images	CNN—ResNet	Accuracy 96.26%		
				Specificity 98.34%		
				Sensitivity 91.86%		
				AUC 0.9875		
Jian Lv et al. [67]	2021	1089 images	CNN—ResNet	Accuracy 96.5%		Grad-CAM and guided Grad-CAM to generate explanation maps and pixel explanations.
				Sensitivity 93.6%		
				Specificity 98.2%		
				AUC 0.983		
Ningning Tang et al. [71]	2023	3364 images	CNN—ResNet	* Fusarium *	* Aspergillus *	Decision tree classifier and CNN-based classifier Grad-CAM and guided Grad-CAM to generate explanation maps and pixel explanation.
				AUC 0.887	AUC 0.827	
Ningning Tang et al. [69]	2023	7957 images	CNN—Inception-ResNet V2 and K nearest neighbor	Precision 90.96%		Two classifiers (CNN- and KNN-based) and two hybrid strategies (weighted voting method and LightGBM) were used to fuse the results.
				Recall 91.45%		
				F1 score 91.11%		
				AUC 0.9841		
Liu Zhi et al. [29]	2020	1213 images	CNN—AlexNet and VGGNet	Accuracy 99.95%		Sub-area contrast stretching algorithm and histogram matching fusion algorithm.
				Sensitivity 99.90%		
				Specificity 100%		
Fan Xu et al. [68]	2021	3453 images	CNN—Inception-ResNet V2	Activated dendritic cells	Inflammatory cells	Transfer learning technique.
				Accuracy 93.19%	Accuracy 97.67%	
				Sensitivity 81.71%	Sensitivity 91.74%	
				Specificity 95.17%	Specificity 99.31%	
				G mean 88.72%	G mean 95.45%	
				AUC 0.9646	AUC 0.9901	

**Table 2:** Summary table for the different DL systems in the detection of dry eye disease when using IVCM images. The performance parameter types depended on the authors’ choices, and the results of the best proposed approaches are detailed. Additional techniques and novelties are also featured.

Authors	Year	Dataset	Artificial Intelligence Method	Results		Additional Techniques and Novelties
Yulin Yan et al. [74]	2023	19,612 images	CNN–ResNet50	Internal test: Accuracy 91.4%, 95.7%, 96.7%, and 95% for the recognition of each layer. Accuracy 96.1%, 93.2%, 94.5%, and 95.9% for normal/abnormal images recognition (for each layer).		
				External test: Accuracy 96.0%, 96.5%, 96.6%, and 96.4% for the recognition of each layer. Accuracy 98.3%, 97.2%, 94.0%, and 98.2% for normal/abnormal image recognition (for each layer).		
Shanshan Wei et al. [88]	2020	5221 images	CNN—ResNet34	AUC 0.96		CNS-Net established.
Dalan Jing et al. [90]	2022	~2290 images	CNN—CNS-Net	The corneal nerve morphology (the average density and maximum length) were significantly correlated with the corneal intrinsic aberrations.		The corneal sub-basal nerve morphology and corneal intrinsic aberrations were investigated with CNS-Net.
Gairik Kundu et al. [93]	2022	120 images	CCMetrics for nerve fiber characteristics and Random Forest classifier	AUC 0.736 Accuracy 86% F1 score 85.9% Precision 85.6% Recall 86.3%		Correlation investigation was conducted between the various clinical symptoms and imaging parameters of ocular surface pain.
Yitian Zhao et al. [94]	2020	322 images	CS-NET The infinite perimeter active contour with hybrid region	Accuracy 81.8% for the first dataset. Accuracy 87.5% for the second dataset.		A Retinex model advanced exponential curvature estimation method with a linear support vector machine.
Baikai Ma et al. [96]	2021	1501 images	kNN-DOWA The infinite perimeter active contour with hybrid region information	The tortuosity was higher in patients with DED than in healthy volunteers ( * p * < 0.001). The tortuosity was positively correlated with the ocular surface disease index ( * r * = 0.418, * p * = 0.003) and negatively correlated with tear breakup time ( * r * = −0.398 and * p * = 0.007). No correlation was found between the tortuosity and visual analog scale scores, corneal fluorescein staining scores, or the Schirmer I test.		
Fernandez et al. [97]	2022	43 images	Watershed algorithm	The tortuosity index was significantly higher in post-LASIK patients with ocular pain than in the control patients. No significant differences were detected with manual measurements. The tortuosity quantification was positively correlated with the ocular surface disease index (OSDI) and a numeric rating scale (NRS) assessing pain.		
Ye-Ye Zhang et al. [98]	2021	8311 images	CNN—DenseNet169	OMGD	AMGD	
				AUC 97.3% Sensitivity 88.8% Specificity 95.4%	AUC 98.6% Sensitivity 89.4% Specificity 98.4%	
Sachiko Maruoka et al. [99]	2020	380 images	CNNs—DenseNet-201, VGG16, DenseNet-169, and InceptionV3	The single DL model: AUC 0.966 Sensitivity 94.2% Specificity 82.1% The ensemble DL model (VGG16 + DenseNet-169 + DenseNet-201 + InceptionV3) AUC 0.981 Sensitivity 92.1% Specificity 98.8%		Transfer learning.
Harry Levine et. al. [101]	2023	173 images	CNNs—CSPDarknet53 and YOLOv3	The mean number of aDCs in the central cornea were quantified automatically: 0.83 ± 1.33 cells/image. The mean number of aDCs in the central cornea were quantified manually: 1.03 ± 1.65 cells/image.		Transfer learning.
Md Asif Khan Setu et al. [102]	2022	1219 images	CNN—U-Net and Mask R-CNN	The CNFs model	The DCs model	
				Sensitivity 86.1% Specificity 90.1%	Precision 89.37% Recall 94.43% F_1_ score 91.83%	

Abbreviations: CNS-Net—corneal nerve segmentation network; LASIK—laser-assisted in situ keratomileusis; aDCs—activated dendritic cells; CNFs—corneal nerve fibers; DCs—dendritic cells.

**Table 3:** Summary table for the different DL systems in the detection of diabetic corneal neuropathy when using IVCM. The performance parameter types depended on the authors’ choice, and the results of the best proposed approaches are shown. Additional techniques and novelties are featured.

Authors	Year	Dataset	Artificial Intelligence Method	Results				Additional Techniques and Novelties
Dabbah et al. [105]	2010	525 images	2D Gabor wavelet and a Gaussian envelope	The automatic analysis is consistent with the manual analysis at a correlation of (r = 0.92).				
Dabbah et al. [106]	2011	521 images	2D Gabor wavelet and a Gaussian envelope	The model had the lowest equal error rate of 15.44%.				
Ioannis N. Petropoulos et al. [107]	2014	186 patients	2D Gabor wavelet and a Gaussian envelope	The manual and automated analysis methods were highly correlated for the following: CNFD (r = 0.9, *p* < 0.0001) CNFL (r = 0.89, *p* < 0.0001) CNBD (r = 0.75, *p* < 0.0001)				
Xin Chen et al. [108]	2017	888 images	2D Gabor wavelet and a Gaussian envelope with dual-tree complex wavelet transforms	Nerve fiber detection: Sensitivity 91.7% Specificity 91.3%				
Xin Chen et al. [109]	2018	176 patients	2D Gabor wavelet and a Gaussian envelope with dual-tree complex wavelet transforms	The AUC for identifying DSPN were comparable: 0.77 for automated CNFD 0.74 for automated CNFL 0.69 for automated CNBD 0.74 for automated ACNFrD.				
Wei Tang et al. [110]	2023	524 images	CNN—MLFGNet	Dice coefficients were 89.33%, 89.41%, and 88.29%.				A multiscale progressive guidance module, a local feature-guided attention module, and a multiscale deep supervision module.
Tooba Salahouddin et al. [112]	2021	108 patients	CNN—U-Net	DPN from the control subjects: AUC 0.86 Sensitivity 84% Specificity 71%				
				DPN from the DPN+: AUC 0.95 Sensitivity 92% Specificity 80%				
				Control subjects from the DPN+: AUC 1.0 Sensitivity 100% Specificity 95%				
Yanda Meng et al. [113]	2023	279 patients	CNN—ResNet50	Sensitivity 91% Specificity 93% AUC 0.95				Grad-CAM and guided Grad-CAM to generate explanation maps and pixel explanations.
Yanda Meng et al. [114]	2022	228 patients	CNN—ResNet50	HV	PN−		PN+	Grad-CAM and guided Grad-CAM to generate explanation maps and pixel explanations.Occlusion sensitivity.
				Recall 100% Precision 83% F1 score 91%	Recall 85% Precision 92% F1 score 88%		Recall 83% Precision 100% F1 score 91%	
Williams et al. [115]	2020	1698 images	CNN—U-Net	Intraclass correlations: Total corneal nerve fiber length 0.933 Mean length per segment 0.656 Number of branch points 0.891				
Erdost Yıldız et al. [116]	2021	510 images	CNN—U-Net and GAN	U-Net		GAN		
				AUC 0.8934		AUC 0.9439		
Guangxu Li et al. [117]	2022	30 images sets	CNN—VGGNet	The stitching method can evaluate the corneal nerve of patients more accurately and reliably compared to a single image.				
Abdulhakim Elbita et al. [118]	2014	356 images	Back propagation neural network	Accuracy 99.4%				DCT filter, Gaussian smoothing, contrast standardized, and Otsu’s threshold.

Abbreviations: CNFD—corneal nerve fiber density; CNFL—corneal nerve fiber length; CNBD—corneal nerve fiber branch density; DSPN—diabetic sensorimotor polyneuropathy; ACNFrD—corneal nerve fiber fractal dimension; DPN—diabetic peripheral neuropathy; HV—healthy volunteer; PN—neuropathy.

## Data Availability

Not applicable.
